# Optimizing Surgical Management of Anterior Skull Base Meningiomas: Imaging Modalities, Key Surgical Considerations, and Risk Mitigation Strategies

**DOI:** 10.3390/cancers17060987

**Published:** 2025-03-14

**Authors:** Gheorghe Ungureanu, Larisa-Nicoleta Serban, Stefan-Ioan Florian

**Affiliations:** Department of Neurosciences, “Iuliu Hatieganu” University of Medicine and Pharmacy Cluj, 400347 Cluj-Napoca, Romania; larisa_serban@yahoo.com (L.-N.S.); stefanfloriannch@gmail.com (S.-I.F.)

**Keywords:** meningioma, cranial fossa, anterior, skull base neoplasms

## Abstract

Skull base meningiomas present considerable challenges in surgical management due to their proximity to critical neurovascular structures. Anterior skull base meningiomas encompass olfactory groove, supra- and parasellar, anterior sphenoid ridge, cavernous sinus, and spheno-orbital tumors. The success of surgical resection and the likelihood of complications are influenced by several key factors, including the presence of an intact arachnoid plane, tumor size and consistency, peritumoral brain edema, cranial nerve involvement, vascular encasement, and invasion of critical areas such as the optic canal or cavernous sinus. These factors not only affect the feasibility of gross total resection but also play a pivotal role in determining functional outcomes and postoperative recovery. With the vast array of imaging modalities available, selecting the most appropriate investigations to assess these parameters and tailoring surgical strategies accordingly remain complex tasks. This review examines the critical surgical parameters, identifies the most effective imaging modalities for evaluating each, and provides key insights into how this analysis can guide surgical decision-making, mitigate risks, and minimize complications.

## Simple Summary

Skull base meningiomas pose significant surgical challenges due to their proximity to vital neurovascular structures. Key factors influencing surgical outcomes include the integrity of the arachnoid plane, tumor size and consistency, brain edema, nerve involvement, vascular encasement, and invasion of critical areas. These factors affect the feasibility of complete tumor removal and postoperative recovery. This review explores critical surgical considerations, effective imaging techniques for evaluation, and strategies to optimize decision-making, reduce risks, and minimize complications.

## 1. Introduction

Anterior skull base meningiomas (ASBM) are among the most complex intracranial tumors to treat, owing to their proximity to critical neurovascular structures and their intricate anatomical locations. These tumors are broadly classified into two primary groups: midline meningiomas, which include ophthalmic groove meningiomas (OGM), planum sphenoidale meningiomas (PSM), tuberculum sellae meningiomas (TSM), and diaphragma sellae meningiomas (DSM); and paramedian meningiomas, encompassing clinoidal and allar meningiomas. Both groups may exhibit extensions toward the orbit and/or cavernous sinus [1]. Each of these anatomical locations presents unique characteristics in terms of neurovascular relationships, growth patterns, and extensions, necessitating distinct surgical strategies tailored to their specific challenges. Key factors influencing surgical feasibility and outcomes include the presence of an intact arachnoid plane, tumor size and consistency, peritumoral brain edema (PTBE), tumor size, optic canal and cavernous sinus involvement, and vascular encasement [2,3,4]. These features impact the success of gross total resection (GTR) as well as complication rates, functional outcomes, and recovery.

Advances in imaging techniques have improved the preoperative assessment of skull base meningiomas by providing detailed information on tumor characteristics and their relationships with surrounding structures [5]. However, the wide variety of available imaging modalities, each with specific benefits and limitations, can make selecting the most suitable investigations challenging [6]. A systematic integration of radiological findings into surgical planning is essential for optimizing strategies, assessing risks, and improving patient counseling regarding potential outcomes [7].

This review outlines a systematic approach to evaluating the key radiological features of ASBM, emphasizing tumor origin, cerebrovascular involvement, resection feasibility, and potential risks. Each of the first seven sections examines a specific tumor trait, its clinical significance, optimal imaging modalities, and assessment strategies. Surgical insights are drawn from existing literature and the senior author’s experience (ISF) with over 500 transcranially operated ASBM cases. The final section compares open and endoscopic approaches, highlighting how radiological findings can guide surgical decision-making and optimize treatment strategies.

## 2. Assessment of Tumor Origin and the Presence of an Arachnoid Cleavage Plane

### 2.1. Surgical Significance

**Figure 1:**
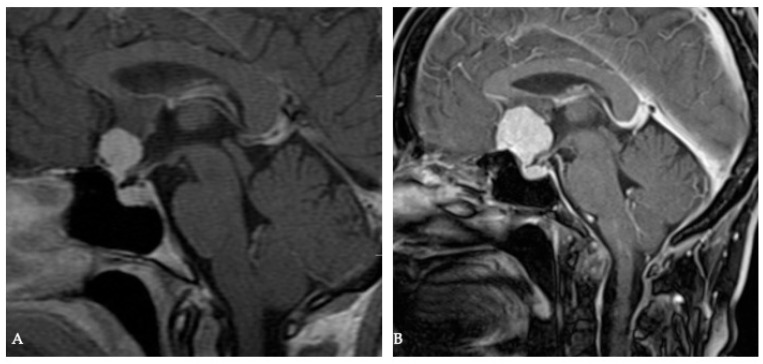
Meningiomas develop along the path of least resistance. A 53-year-old female presented in 2013 with an incidental finding of meningioma of the tuberculum sellae (**A**). She refused surgical treatment. In 2017 she presented with visual disturbance in the right eye. The MRI outlined the growth of the tumor (**B**). Meningiomas of the tuberculum sellae are restrained by the arachnoid covering the ICA and posterior communicating arteries, optic nerves, optic chiasm, lamina terminalis, pituitary stalk, infundibulum, and Lilequist membrane. Consequently, the only route for tumor spread is anteriorly over the planum, over the optic nerves, and above the chiasm around the anterior cerebral artery complex. The growth over the planum may be the result of an anatomic defect in the arachnoid of the chiasmatic cistern at the chiasmatic sulcus or a postfixed chiasm.

The presence of an arachnoidal cleavage plane is consistently recognized as one of the most critical factors for predicting the feasibility of safe surgical intervention and the likelihood of achieving gross-total resection (GTR), irrespective of tumor location or the chosen surgical approach [8,9,10,11,12,13,14,15,16,17,18]. Preserving the arachnoid directly influences functional outcomes and can facilitate functional preservation or improvement [19,20]. The origin of an ASBM will determine how the tumor will displace the cerebrovascular structures and the arachnoid surrounding them. Skull base meningiomas grow following the “path of least resistance” [12,21]. This is supported by the observation that meningiomas originating in a specific anatomical location generally demonstrate consistent morphologies across patients, with reliably predictable growth trajectories in those monitored through serial imaging [22] (Figure 1).

The pattern of arachnoid displacement is consistent among tumors arising from the same location, making precise determination of the tumor’s origin an important surgical consideration [12,21]. Previous studies have shown that incorporating growth and extension patterns can differentiate tumors arising in proximity—for example, distinguishing clinoidal meningiomas from inner sphenoid wing meningiomas [9,23].

Thus, assessing the tumor’s origin in conjunction with evaluating the presence of an arachnoidal cleavage plane is critical for preoperative planning. Even if a distinct CSF plane is not readily visible, the tumor’s location can guide expectations regarding the existence of an arachnoid membrane. Integrating these factors enables a more accurate assessment of the feasibility and safety of surgical resection and risk evaluation for cerebrovascular structures.

### 2.2. Imaging Evaluation

**Figure 2:**
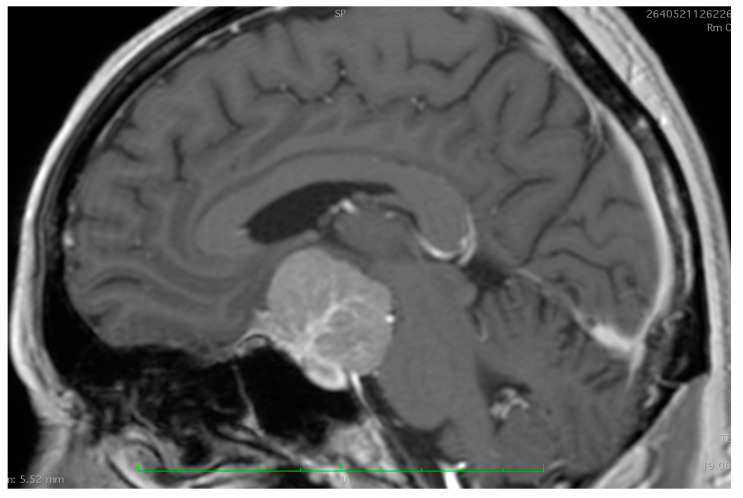
CE-T1WI Sagittal view of a skull base meningioma. The pattern of vascular supply and dural enhancement suggests that the origin of the tumor is the tuberculum sellae.

The tumor’s origin can be effectively assessed using sagittal contrast-enhanced T1-weighted imaging (CE-T1WI), where the dural base is typically visualized as a thickened structure with intense enhancement. Furthermore, sagittal CE-T1WI or T2-weighted imaging (T2WI) can reveal the “sunburst” sign or a distinctive vascular pattern originating from the tumor, providing additional diagnostic insights [24] (Figure 2). CT imaging is particularly valuable for detecting hyperostosis, which can serve as an indication of the tumor’s origin and is a feature associated with an increased risk of recurrence, thus justifying more aggressive surgical approaches when feasible [25,26,27]. The information gained from DSA is readily available by less invasive methods, and its use should be limited to cases in which a preoperative embolization is contemplated [24].

For identifying an arachnoidal cleavage plane, CISS (constructive interference in steady state) MRI imaging with multiplanar reconstructions is the preferred modality [28]. This imaging modality allows for visualization of the direction of membrane displacement, and in cases other than giant meningiomas, it can help infer the presence of an arachnoid plane protecting the vessels [29,30]. CISS imaging strongly correlates with intraoperative findings of meningioma dural attachment, with dural irregularities indicating tumor origin and feeding vessel location, which can guide the surgeon for tumor devascularization [31].

### 2.3. Key Surgical Considerations

**Figure 3:**
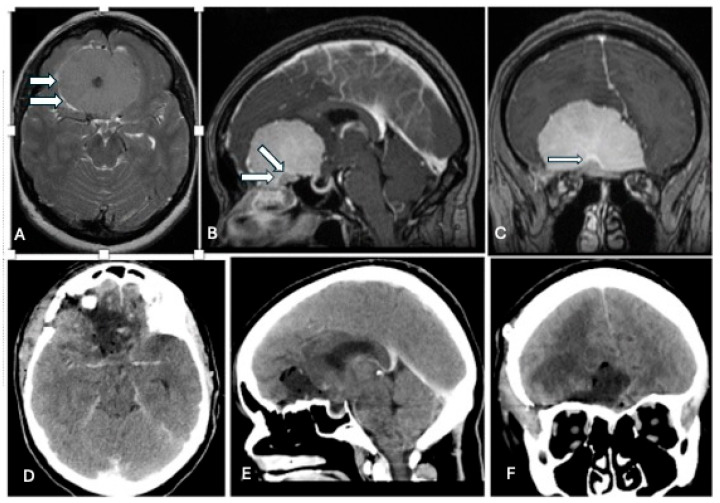
A preoperative CE-T1WI and postoperative CT of a large ophthalmic groove meningioma (**A**–**C**), which was completely resected via a right frontolateral approach (**D**–**F**). Note the presence of an arachnoidal cleavage plane (image (**A**) arrow) and dural enhancement at the level of the ophthalmic groove (images (**B**,**C**), arrows).

○ Basal devascularization: Initiating resection with basal devascularization at the tumor’s origin is a critical first step to achieving complete resection.
○ Extradural devascularization: For clinoid and alar meningiomas approached through a frontolateral or pterional approach, this technique reduces blood loss and softens the tumor, facilitating removal.
○ Intradural devascularization: In tuberculum and diaphragma sellae meningiomas, this should be performed only after identifying major regional anatomical structures.
○ Follow the arachnoid: The arachnoid plane, its fibrous structures, and compartmentations direct the tumor growth and provide a pathway for tumor resection while protecting vascular and nerve structures covered by their own arachnoid.
○ The choice between a “skull base” or “vascular” approach for these tumors remains a topic of ongoing debate [24]. In cases without significant extradural extension, the frontolateral approach is a highly suitable option for most tumors (Figure 3). It provides an optimal balance of exposure, versatility, and simplicity while minimizing the risks associated with more complex skull base techniques [32].

## 3. Tumor Size

### 3.1. Surgical Significance

Tumor size is frequently cited as a factor affecting the likelihood of achieving gross total resection (GTR) and the choice of surgical approach [15,23,33,34]. While some studies have found an association between tumor size and postoperative outcomes—such as complication rates, visual preservation, or mortality—others have reported no such correlation [23,34,35,36,37,38].

Variability in the reported impact of tumor size on surgical resection and prognosis may stem from its interplay with other factors. For example, tumors with firm consistency are more challenging to resect, whereas the resection of soft tumors may be less dependent on size. Larger tumors tend to disrupt arachnoid membranes, resulting in adherence to cerebrovascular structures, which increases surgical risk and decreases GTR rates. Moreover, large tumors are more likely to extend into multiple compartments, reducing the likelihood of complete resection. They could also potentially cause greater stretching and secondary ischemia of adjacent neural structures, leading to a higher risk of deficits.

### 3.2. Imaging Evaluation

Tumor size is typically reported as the maximum diameter observed on any MRI plane [39,40]. Although tumor volume is the more objective and accurate measure, it may not be as useful in clinical practice [16]. Sekhar proposed the use of tumor equivalent diameter (TED), calculated as the geometric mean of three orthogonal dimensions on CE-T1WI: TED = (D1 × D2 × DE) 1/3 TED = (D1 × D2 × DE) 1/3 [41]. While definitions of small, medium, and large tumors vary in the literature, we recommend classifying tumors based on their largest diameter on CE-T1WI: small (<3 cm), medium (3–5 cm), and large (>5 cm). This standardization aligns with existing definitions across different ASBM locations.

### 3.3. Key Surgical Considerations

○ Tumor size affects both intraoperative and postoperative management.
○ Large meningiomas often show variable consistency: denser at the core/base and softer at the periphery.
○ Larger tumors provide more workspace as volume decreases, allowing multi-directional approaches.
○ Postoperative issues in large meningiomas include regional circulatory changes causing venous hemorrhages, ischemia, or cerebral edema.
○ CSF compensates for the tumor space, potentially causing pseudo meningocele or CSF fistula. Issues are typically managed with lumbar drainage, compressive dressing, and diuretics; persistent hypersecretion may require subgaleo-peritoneal drainage.

## 4. Assessment of Tumor Consistency

### 4.1. Surgical Significance

Tumor firmness is associated with more challenging resections, lower GTR rates, longer operative times, and increased complication risks, particularly when the capsule does not fold during resection [15,42]. These challenges are further amplified in large tumors. While advanced microsurgical tools, such as ultrasonic aspirators, have improved the resection of firm tumors, consistency remains a key factor in surgical decision-making.

### 4.2. Imaging Evaluation

**Figure 4:**
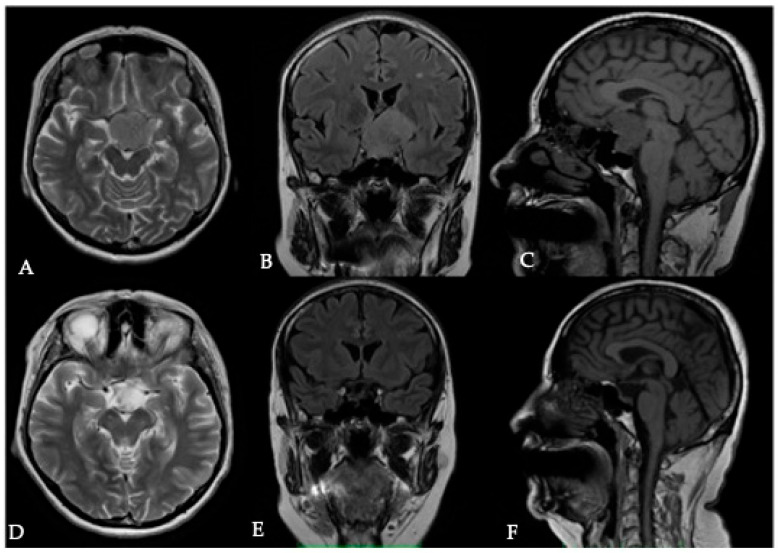
Pre- and postoperative MRI images of a patient with a tuberculum sellae meningioma (TSM). The preoperative images demonstrate hyperintensity on T2-weighted imaging (**A**) and T2 FLAIR (**B**) along with hypointensity in T1WI (**C**), indicative of a soft tumor, which facilitates complete removal. Postoperative MRI sections (**D**–**F**), shown below, confirm complete tumor resection.

CT has not shown reliable effectiveness in predicting tumor consistency in multiple studies [43,44,45]. While some studies report conflicting results, tumor appearance on standard T2WI, FLAIR, and T1WI MRI can reliably predict consistency in most cases when compared to cerebral gray matter. One study showed that T2 hypointensity strongly indicated firm consistency (100%; *p* < 0.001), while T2 hyperintensity combined with T1 hypointensity predicted softness (90%; *p* < 0.01) [45]. Another study confirmed that higher T2WI and FLAIR signal intensity correlated with softer tumors, while hypointense tumors were consistently hard. T2WI and FLAIR were identified as independent predictors of tumor consistency, with softer tumors showing better radical resection rates (lower Simpson grades) [44] (Figure 4). Researchers developed a T2WI-based scale using the tumor-to-cerebral peduncle intensity ratio to classify tumors as soft, moderate, or firm [46].

When using standard MRI sequences to assess tumor consistency, it is important to note that MR intensity values are based on arbitrary units, preventing direct comparisons across acquisitions. Additionally, vendor-specific hardware and pulse sequences can further complicate evaluation [47]. Although MR elastography is specifically developed to evaluate tumor stiffness, its application in current neurosurgical practice remains limited. Moreover, its accuracy and utility in assessing smaller or highly vascular tumors have not yet been fully optimized [48].

### 4.3. Key Surgical Considerations

○ Soft tumors: Associated with a higher chance of GTR. Risks include arterial vessel encasement—avoid arterial trunk coagulation until intratumoral trajectory is clear; strictly coagulate tumor feeders.
○ Firm/calcified tumors: Pose challenges for volume reduction and dissection of adjacent structures; initial basal devascularization is critical, with calcified tumors requiring progressive drilling and immediate sealing of vascular canals with bone wax.
○ In cases where injury to critical structures could lead to significant neurological deficits, leaving a small portion of the tumor on the affected tissue may be a safer approach.

## 5. Assessment of Peritumoral Brain Edema (PTBE)

### 5.1. Surgical Significance

Most studies concur that the presence of PTBE is associated with lower rates of GTR and an increased risk of postoperative complications in skull base meningioma surgeries [18,49,50,51]. Four main theories have been proposed to explain PTBE development in meningiomas [52,53]: (1) The secretory-excretory theory—secretory meningiomas release proteinaceous substances causing osmotic edema. Rare and insufficient to explain most cases. (2) The cerebral compressive theory—larger tumors compress the brain and cause ischemia, but this theory does not account for PTBE in small tumors. (3) The vascular compression theory—venous obstruction is inconsistently linked to increased edema. (4) The hydrodynamic theory—intratumoral congestion triggers angiogenesis, immature vessels, and plasma leakage. However, the exact mechanisms of PTBE remain unclear.

In a study of 696 patients with meningiomas and PTBE, older age and male sex were significantly associated with the presence of PTBE [54]. Olfactory groove meningiomas (OGMs) and tumors near the Sylvian veins are particularly prone to PTBE [54,55]. The relationship between tumor volume and PTBE is debated, with studies showing conflicting results [52,56]. A disrupted arachnoid plane demonstrates the strongest correlation with PTBE, and it is important to note that the surgical difficulties associated with PTBE may be attributed to this disruption [56].

### 5.2. Imaging Evaluation

The presence of PTBE can be assessed by using standard T2WI and FLAIR MRI sequences and on CT when MRI is not available. Radiological factors such as irregular margins, T2 hyperintensity, heterogeneous enhancement, and pial blood supply have been linked to PTBE, but the strength of these associations varies [57,58]. Domingues et al. classified it as light (smaller or equal to the volume of the tumor), moderate (double the volume of the tumor), and severe (more than twice the volume of the tumor) [59]. Some imaging programs provide a tool for volume measurement, and a measuring tool is also available on most neuronavigation software, which is now widely utilized in neurosurgical centers [54,60].

### 5.3. Key Surgical Considerations

○ The presence of PTBE should serve as an indicator of potential arachnoid disruption; exercise greater caution during tumor dissection from surrounding cerebral tissue.
○ Infiltration of the pia mater, with a glial pseudocapsule and tortuous arterialized vessels, suggests a complex PTBE mechanism.
○ Edema is a better indicator of a higher risk of postoperative neurological worsening rather than tumor resectability.
○ A two-staged approach, where an initial surgery leaves a shell of the tumor in place and a second surgery for complete removal is performed after PTBE subsides, has been described for OGMs [55]. Its advantages are largely theoretical, as PTBE may persist for years even after the complete removal of a meningioma.

## 6. Vascular Encasement

### 6.1. Surgical Significance

Vascular injury, particularly involving the ICA, its major branches, and perforators, is a major risk in ASBM. Reported vascular complication rates, likely underreported, range from 1% to 19%, particularly in resource-limited settings [2,61,62,63]. One study noted a 7% intraoperative fatality rate from ICA injuries during extended endoscopic approaches [64]. Neoplastic invasion of the vessel wall makes it especially vulnerable to the mechanical stress exerted during surgery [63,65].

Tumor origin and extension patterns greatly influence interactions with cerebral vasculature. Vessels protected by multiple arachnoid membranes are less prone to injury, underscoring the importance of carefully analyzing these patterns to identify high-risk cases, such as clinoidal meningiomas [8]. Our experience, consistent with prior studies, shows that vessel narrowing is a strong indicator of wall involvement, and excessive mechanical traction during surgery can result in rupture or elevate the risk of postoperative vasospasm [11,23,66].

### 6.2. Imaging Evaluation

**Figure 5:**
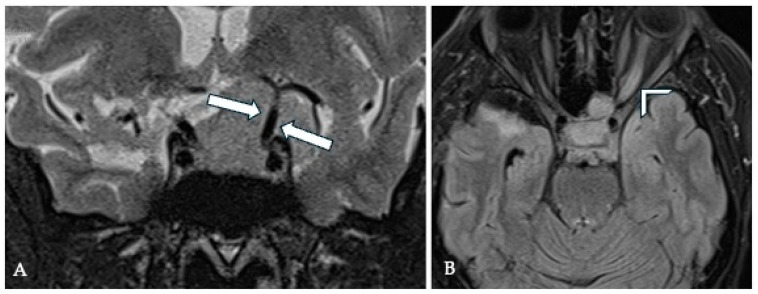
Coronal fine-section T2WI of a patient with a history of TSM resection via right pterional craniotomy at another center, now presenting with left eye vision loss (**A**). Imaging reveals complete encasement of the left ICA and bilateral cavernous sinus involvement (CSI). Hyperintensity surrounding the left ICA indicates the presence of an arachnoid cleavage plane, with no evidence of vessel narrowing (arrow). Axial FLAIR imaging (**B**) demonstrates marked edema of the left optic nerve (ON), correlating with the patient’s preoperative visual impairment (arrowhead).

CISS MRI and fine T2WI demonstrate superior performance compared to MPRAGE, and both modalities are likely more effective than MRA or CTA in providing detailed visualization of tumor-vessel relationships and detecting vessel narrowing [53] (Figure 5). The presence of an arachnoid plane should be systematically assessed. Combining CTA with MRI is extremely useful if neuronavigation is employed. Most information traditionally obtained from DSA can now be derived from non-invasive imaging modalities. However, DSA remains particularly useful in cases where the tumor causes narrowing of the ICA diameter and if balloon testing is required [2,67].

Although no current imaging modality reliably identifies the relation between tumor and perforating vessels, their involvement should be presumed in cases of complete ICA or branch encasement. Emerging techniques such as High-Resolution Compressed-Sensing T1 Black-Blood MRI show promise for better evaluation of vessel wall involvement; however, their clinical utility remains limited at the time of writing [68].

Vessel involvement should be systematically assessed and reported using standardized terminology. A recommended approach includes specifying whether the tumor displaces the vessel without encircling its walls; partially encases the vessel (e.g., 50% or 75%, corresponding to 180–270 degrees); completely encases the vessel (360 degrees); and if it causes narrowing of the vessel. A complete engulfment of the ICA or ACA, especially when no arachnoidal cleavage plane is visible and tumors that extend laterally to the ICA represent contraindications for an endoscopic approach.

### 6.3. Key Surgical Considerations

○ Imaging suggests the tumor’s relationship with adjacent vessels, but intraoperative findings determine resectability without vascular injury.
○ Tumors grow within their own space, displacing or partially encompassing arterial trunks, protected by the arachnoid layer. Progressive debulking allows the delicate mobilization of smaller fragments through windows created by vascular structures.
○ Leave a thin tumor layer if detachment risks vascular damage.
○ Perforating arteries, usually on the tumor surface, can be delicately detached within the arachnoid plane; avoid coagulation near perforator origins to prevent shrinkage.
○ ACI injury causes severe bleeding and obstructs visibility. Temporary vessel clipping enables assessment and action: Direct vascular wall suturing is sometimes possible.
○ Microclip placement for small lateral lacerations to stop bleeding is often more effective than emergency anastomosis. In cases in which this is necessary, the options include termino-terminal anastomosis for the ACA trunk and termino-lateral anastomosis for major arteries arising from the ICA.

## 7. Optic Canal Involvement (OCI) and Optic Nerve (ON) Compression

### 7.1. Surgical Significance

Visual disturbances are among the most common presenting symptoms in patients with supratentorial skull base meningiomas and, as such, are one of the main causes of disability associated with these tumors that need to be addressed through surgery [20,69,70]. Compression of the ON and its subsequent ischemia are the likely causes of the visual impairment [71]. The reported prevalence of tumor invasion in the optic canal varies widely in the literature [72]. OCI and ON involvement will also guide the surgical approach (see Section 9). Whether routine optic canal opening should be performed in cases of suprasellar tumors remains controversial, as studies present conflicting evidence regarding its safety and efficacy [69,73,74]. Authors that support it argue that it is a safe procedure and could ensure a proper decompression of the ON [73,75,76,77,78]. However, studies that do not employ routine optic canal opening report comparable visual outcomes [37,69,79,80]. Additionally, there is no consensus on the optimal timing of optic canal opening, with opinions differing on whether it should be performed before or after tumor resection [16,39,75]. The ONs anchor inner arachnoid membranes from surrounding cisterns, which typically protect them in most supratentorial skull base meningiomas, directly influencing surgical success [20,81,82].

### 7.2. Imaging Evaluation

**Figure 6:**
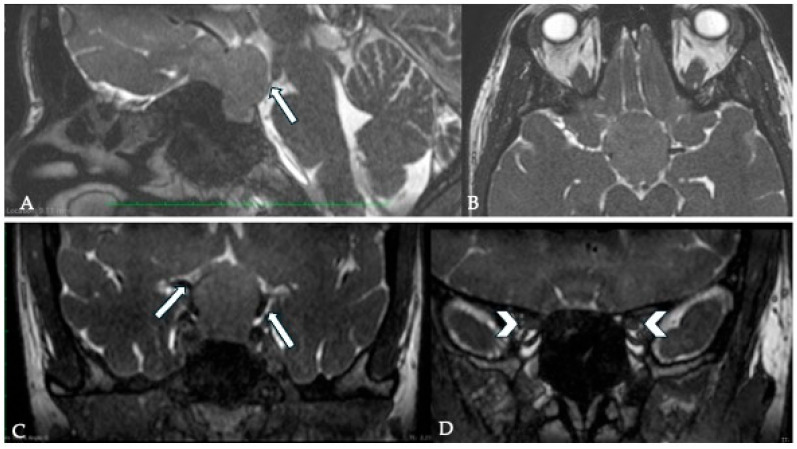
CISS-MRI imaging of a TSM. The sagittal view (**A**) illustrates the tumor’s origin and typical extension pattern over the planum sphenoidale, with the displaced pituitary stalk clearly visible (arrow). The axial view (**B**) reveals ample CSF spaces, indicating preserved arachnoid membranes. A coronal section at the level of the cavernous sinus (**C**) shows bilateral displacement of the ICAs and upward displacement of the ACA complex (arrows). A coronal section at the clinoid level (**D**) demonstrates no optic canal invasion (OCI), with the optic nerve (ON) clearly visible and surrounded by CSF (arrowheads).

OCI should be assessed using fine-cut CE-T1 and T2WI in coronal views, dividing the OC into four quadrants for invasion evaluation [77,83,84,85]. MRI hyperintensity of the ON correlates strongly with preoperative and postoperative visual disturbance [29] (Figure 5). The optic apparatus is most effectively visualized with CISS imaging (Figure 6). A comparison showed CE-CISS provided superior visualization of anterior optic pathways (100% vs. 78% with conventional MR imaging), predicted persistent visual impairment with 75% sensitivity and 96% specificity when hyperintensity was present post-surgery, and assessed tumor adherence to optic pathways, aiding in tumor dissection feasibility [29,86]. The feasibility of an endonasal endoscopic approach should be carefully assessed based on the specific quadrants of the OC affected by the tumor and its spatial relationship to the ON. While some studies indicate that MRI identifies only approximately 65% of cases with OCI, others suggest that in the absence of imaging evidence of invasion, surgical opening may not be necessary [69,73,77,83].

### 7.3. Key Surgical Considerations

○ The arachnoid at the interface with the ON should be preserved during surgery, as this has been linked to an improved visual recovery after surgery [20].
○ Opening of the OC is essential for tumor extension into it but should be avoided routinely to prevent unnecessary complications.
○ A bulging optic nerve suggests a fragment beneath it and warrants further inspection.
○ Gentle canal opening enables detachment of intracanal tumor fragments, guided by the preserved arachnoid plane.
○ Prolonged compression may erode the arachnoid layers, requiring canal opening and tumor mass reduction to protect the ON.

## 8. Cavernous Sinus Involvement (CSI)

### 8.1. Surgical Significance

Incomplete resection of a meningioma is associated with a higher risk of recurrence, and as such, portions of the tumor growing in the cavernous sinus (CS) could lead to a higher chance of relapse in cases of incomplete resections. Over the past 30 years, the treatment of CS meningiomas has evolved from aggressive surgical resection, despite high complication rates, to a more conservative approach. This shift reflects a better understanding of tumor behavior and the significant morbidity associated with extensive surgery [87,88,89].

The advent and widespread adoption of stereotactic radiotherapy and radiosurgery (SRS) have further refined treatment strategies. These modalities provide excellent tumor control, with progression-free survival (PFS) rates exceeding 90% at 10 years, and are associated with low complication rates [90,91,92]. As a result, indications for surgical treatment of these lesions have become highly limited. Currently, surgical intervention is typically confined to the extracavernous portion of the tumor, with aggressive resection reserved for exceptionally rare cases [93,94]. Proper identification of cases requiring surgical resection is critical, as such interventions can negatively impact the outcomes of subsequent stereotactic radiosurgery [95]. Thorough evaluation of CSI and its relationship with cranial nerves (CN) and the ICA’s is crucial for accurately counseling patients on surgical, radiosurgical, or conservative management risks [93].

### 8.2. Imaging Evaluation

Studies suggest CSI is best visualized on T2WI or CISS compared to CE-T1WI, with CISS particularly useful for delineating tumor boundaries and its relationship with cranial nerves, aiding in surgical and SRS planning [29,96,97,98,99]. When the tumor displaces the ICA and cranial nerves medially without significant encasement (extracavernous tumor), surgical intervention may be a viable treatment option [93]. The Knosp grading system, originally for pituitary adenomas invading the cavernous sinus, helps characterize this relationship [100]. Post-contrast T1WI and TOF imaging are valuable for delineating the tumor’s relationship with the internal carotid artery (ICA), assessing encasement, and identifying narrowing.

**Figure 7:**
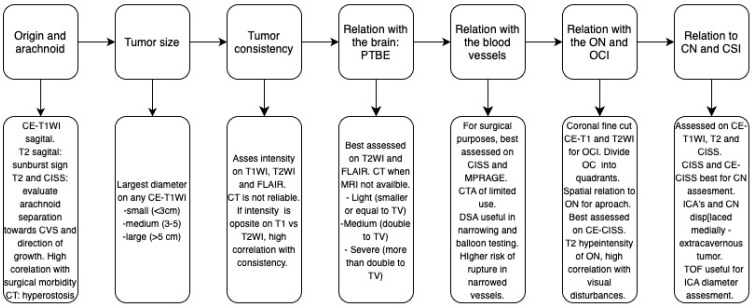
Summary of the main characteristics of anterior skull base meningiomas (ASBMs), preferred imaging modalities, and their key imaging features. This figure serves as a practical reference for selecting the most appropriate imaging approach based on tumor characteristics. CVS—cerebrovascular structures; TV—tumor volume.

CT scans are useful for the assessment of hyperostosis and should be reserved only for cases in which surgery is contemplated to limit irradiation [93]. Similarly, DSA should only be performed in cases where intracavernous surgery is contemplated, to better assess stenosis and to perform a balloon occlusion test, as the artery is at a high risk of rupture in surgery for CSI [67,89,94]. Figure 7 presents a step-by-step diagram of tumor characteristics and preferred imaging modalities.

### 8.3. Key Surgical Considerations

○ Complete resection of intracavernous meningiomas is risky due to cranial nerve injury, ICA laceration, and cavernous sinus bleeding.
○ Extracavernous portion reduction suffices for optimal neurological outcomes.
○ Decompression via tumor tracking into the cavernous sinus relieves pain.

## 9. Importance of Preoperative Imaging and Clinical Characteristics for Approach Selection

A detailed comparison of surgical approaches for ASBM is beyond this article’s scope, but key factors guiding approach selection are worth noting. Extended endonasal approaches (EEA) are widely used, yet no randomized trials definitively compared them with microscopic transcranial approaches (mTCA). Proposed classification scales aim to guide selection, but inconsistencies in what they consider important limit their clinical applicability [16,101,102]. As each technique has different indications, selection bias influences approach choice, and much of the supporting literature relies on expert opinions that do not reflect the broader neurosurgical community experience and expertise [7,103,104].

Several meta-analyses and systematic reviews have compared EEA and mTCA for midline ASBM [61,105,106,107]. A comparison between the results of the two surgical attitudes is presented in Table 1. It is important to mention that results for mTCA vary greatly between various surgical approaches. Findings suggest that EEA offers superior visual outcomes but carries a higher risk of CSF fistulas. However, the incidence of this complication has significantly decreased over the past 25 years, particularly in cases requiring revision surgery or those associated with severe infections [108,109]. In contrast, mTCA is more effective in preserving olfaction when it is still intact in OGM. Additionally, studies indicate that EEA is most suitable for tumors that do not extend beyond certain lateral landmarks, such as the ON and ICA’s, and that do not completely encase cerebral vessels [110,111,112,113,114,115].

**Figure 8:**
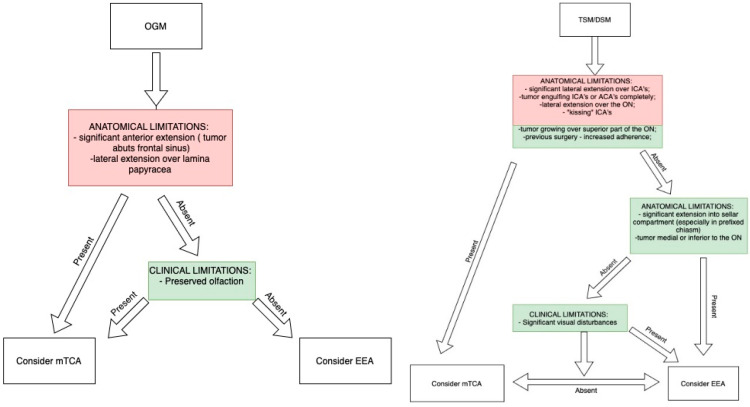
Decision-making algorithm for selecting the appropriate approach based on preoperative imaging and clinical characteristics. Boxes in red represent absolute contraindications, and those in green relative contraindications.

As some authors emphasize, the choice between EEA and mTCA is not a matter of overall superiority but rather an assessment of their respective advantages and limitations. The optimal approach should be tailored to the individual patient, taking into account tumor characteristics and the surgeon’s expertise and experience [116]. Furthermore, a combination of the two approaches can offer several benefits in complex tumors [117]. A decision-making algorithm, incorporating radiological characteristics discussed in this article along with relevant clinical factors, is presented in Figure 8.

While EEA provides an alternative approach to mTCA for midline tumors, its use has been limited in paramedian meningiomas. In these cases, the use of an endoscopic transorbital approach (eTOA) has gained traction. This approach has been described for pathologies of the orbit, anterior and middle cranial fossae, petrous apex, and cavernous sinus [118]. This approach seems to be of value in cases with ACBM with CSI in which proptosis is a main clinical symptom [119].

## 10. Conclusions

Anterior skull base meningiomas (ASBM) present substantial surgical challenges due to their complex anatomy and proximity to critical neurovascular structures. This review underscores the indispensable role of systematic radiological assessment in optimizing surgical planning and enhancing patient outcomes. Key imaging parameters—such as tumor origin, presence of an arachnoid cleavage plane, consistency, size, peritumoral edema, vascular encasement, and optic canal involvement—are crucial in determining the feasibility and safety of GTR. Integrating imaging findings into surgical planning, alongside a comprehensive understanding of tumor-specific factors, continues to refine the treatment paradigm for ASBM.

High-resolution MRI techniques, including CISS, CE-CISS, and fine-cut T2-weighted imaging, are particularly effective in delineating the arachnoidal cleavage plane, evaluating tumor–vascular relationships, assessing optic canal involvement, and determining cranial nerve encasement. For tumor consistency assessment, a combination of T1WI, T2WI, and FLAIR sequences provides valuable predictive information, while CE-T1WI remains fundamental for determining tumor origin, size, and cerebrospinal invasion. The essential surgical principles of meningioma resection—the “4Ds” (debulking, devascularization, dissection, and detachment)—can largely be anticipated through careful MRI analysis. Tumor consistency predicts debulking feasibility, while tumor origin guides intradural vs. extradural devascularization. Dissection depends on identifying an intact arachnoid plane, and detachment is influenced by both the arachnoidal plane and tumor consistency. TOF aids in vascular assessment, CT is instrumental in detecting hyperostosis, and digital subtraction angiography (DSA) remains important for evaluating vascular encasement when balloon occlusion testing is required. The following key surgical principles emerge:

Basal and Extradural Devascularization: Initiate resection with basal devascularization. For clinoid and alar meningiomas, extradural devascularization minimizes blood loss and softens the tumor.
Intradural Devascularization: Crucial for tuberculum and diaphragma sellae meningiomas after identifying key anatomical structures.
Arachnoid-Guided Resection: Following the arachnoid plane protects critical vascular and nerve structures.
Approach Selection: The frontolateral approach balances exposure and safety for tumors without significant extradural extension. The EEA is best for visual preservation in tumors without extensive lateral extension and without vascular encasement.
Tumor Size: Larger tumors allow multi-directional resection but pose postoperative risks like edema and CSF leaks.
Soft vs. Firm Tumors: Soft tumors have higher GTR rates but a higher risk of vessel encasement; firm/calcified tumors require patience, drilling, patience and cautious dissection.
Peritumoral Brain Edema (PTBE): Indicates potential arachnoid disruption and increased risk of neurological deficits. Staged resection may be considered.
Vascular Considerations: Tumors displace rather than invade vessels. Preserve the arachnoid layer, debulk progressively, and avoid excessive coagulation near perforators.
Optic Nerve Protection: Preserve the arachnoid interface for better visual outcomes; open the optic canal only when necessary.
Cavernous Sinus Involvement: Total resection is high-risk; extracavernous reduction suffices for symptom relief.

By integrating these imaging tools into a structured preoperative workflow, neurosurgeons can refine their approach, improving both the safety and efficacy of ASBM resection. Future studies should aim at a faster integration of new MRI techniques (MR elastography and black-blood MRI) into the clinical care of patients with ASBM, offering clear protocols for radiologists on how these techniques should be applied in these cases. While RCTs comparing mTCA with EEA are highly unlikely, more interinstitutional studies with homogenous inclusion criteria for patients assigned to one approach or the other could represent a first step towards drafting better indications and could also allow for a better comparison between the two in the larger neurosurgical community. The use of a combination of approaches, the integration of new surgical techniques (e.g., eTOA), could represent the next significant improvement in the surgical care of patients with ASBM.

## Figures and Tables

**Table 1:** Comparison of various outcome metrics for EEA and mTCA. NA: not applicable.

	OGM		TSM	
	EEA	mTCA	EEA	mTCA
GTR	70–80.7%	84.7–98.1%	79–88%	87–92%
30-day mortality	0–4.2%	0.3–3.9%	0–1.7%	0–1.7%
Visual Improvement	64–87%	12–82.2%	71.9–92%	57.9–71.9%
Olfaction Preservation	0%	17.8–29%	NA	NA
CSF Leak	14.4–25.7%	1.6–10.5%	5.3–13.1	0–5.5%
